# *Chd8* mutation in oligodendrocytes alters microstructure and functional connectivity in the mouse brain

**DOI:** 10.1186/s13041-020-00699-x

**Published:** 2020-11-23

**Authors:** Atsuki Kawamura, Yoshifumi Abe, Fumiko Seki, Yuta Katayama, Masaaki Nishiyama, Norio Takata, Kenji F. Tanaka, Hideyuki Okano, Keiichi I. Nakayama

**Affiliations:** 1 Department of Molecular and Cellular Biology, Medical Institute of Bioregulation, Kyushu University, 3-1-1 Maidashi, Higashi-ku, Fukuoka, Fukuoka 812-8582 Japan; 2 Department of Neuropsychiatry, Keio University School of Medicine, Shinjuku, Tokyo 160-8582 Japan; 3 Department of Physiology, Keio University School of Medicine, Shinjuku, Tokyo 160-8582 Japan; 4 Live Imaging Center, Central Institute for Experimental Animals, Kawasaki, Kanagawa 210-0821 Japan

**Keywords:** Autism spectrum disorder, CHD8, Oligodendrocyte, Diffusion tensor imaging, Functional connectivity

## Abstract

*CHD8* encodes a chromatin-remodeling factor and is one of the most recurrently mutated genes in individuals with autism spectrum disorder (ASD). Although we have recently shown that mice heterozygous for *Chd8* mutation manifest myelination defects and ASD-like behaviors, the detailed mechanisms underlying ASD pathogenesis have remained unclear. Here we performed diffusion tensor imaging (DTI) and resting-state functional magnetic resonance imaging (rsfMRI) in oligodendrocyte lineage-specific *Chd8* heterozygous mutant mice. DTI revealed that ablation of *Chd8* specifically in oligodendrocytes of mice was associated with microstructural changes of specific brain regions including the cortex and striatum. The extent of these changes in white matter including the corpus callosum and fornix was correlated with total contact time in the reciprocal social interaction test. Analysis with rsfMRI revealed changes in functional brain connectivity in the mutant mice, and the extent of such changes in the cortex, hippocampus, and amygdala was also correlated with the change in social interaction. Our results thus suggest that changes in brain microstructure and functional connectivity induced by oligodendrocyte dysfunction might underlie altered social interaction in mice with oligodendrocyte-specific CHD8 haploinsufficiency.

## Introduction

Autism spectrum disorder (ASD) encompasses a range of neurodevelopmental disorders characterized by deficits in social interaction and communication as well as by restricted and repetitive behaviors. Structural and functional alterations in the brain of individuals with ASD have been identified by magnetic resonance imaging (MRI). For example, volumetric differences in several brain regions including the cortex, striatum, amygdala, and cerebellum have been detected in such individuals [1, 2]. Diffusion tensor imaging (DTI), a specific type of MRI, has also revealed microstructural changes in gray and white matter that are associated with ASD [3]. Moreover, resting-state functional MRI (rsfMRI) has uncovered a complex pattern of both hypo- and hyperconnectivity across the brain of ASD patients [4, 5]. Whether the alterations detected by such imaging studies contribute to the symptoms of ASD has remained to be demonstrated, however.

Mutations in the gene for chromodomain helicase DNA-binding protein 8 (CHD8) have been identified as a highly penetrant risk factor for ASD by exome-sequencing studies [6–9]. CHD8 is a member of the CHD family of enzymes that belong to the SNF2 superfamily of ATP-dependent chromatin remodelers, and it has been shown to regulate the expression of developmental and ASD risk genes including those related to synaptic function and neurogenesis [10–13]. In humans, *CHD8* mutations give rise to core ASD phenotypes as well as macrocephaly and gastrointestinal complaints [14]. MRI of individuals with *CHD8* mutations has revealed white matter defects, ventriculomegaly, and unspecified abnormalities [14–16]. Furthermore, we and others have found that heterozygous deletion of *Chd8* in mice results in altered social behavior, increased anxiety-like behavior, and cognitive deficits reminiscent of those seen in humans with *CHD8* mutations [17–21]. *Chd8* mutant mice are thus a powerful tool for investigation of the pathogenesis of ASD.

MRI is a key method for translational research to bridge the gap between patients and animal models. In addition to MRI evaluation of ASD patients, recent studies have identified structural and functional alterations across the brain in several mouse models of ASD. One such mouse model, the BTBR T + tf/J mouse, manifests volumetric and structural changes in the striatum, nucleus accumbens, thalamus, and amygdala that were found to be correlated with changes in sociability and repetitive behaviors [22, 23]. Several genetic mouse models of ASD also manifest altered functional connectivity among various brain regions [24–29]. MRI studies in *Chd8* heterozygous mutant mice have thus suggested the presence of microstructural changes in several brain regions as well as functional overconnectivity in the cortico-hippocampal network [19, 20]. Although we recently found that CHD8 haploinsufficiency in oligodendrocytes results in myelination defects and delayed action potential propagation as well as recapitulates certain behavioral phenotypes of *Chd8* heterozygous mutant mice [30], whether oligodendrocyte dysfunction induced by *Chd8* mutation gives rise to such structural and functional changes in the brain has remained unclear.

We have now searched for structural and functional changes in the brain of oligodendrocyte lineage-specific *Chd8* heterozygous mutant mice with the use of MRI. DTI revealed an altered microstructure in some regions of the cortex and striatum in the mutant mice. Furthermore, rsfMRI showed that oligodendrocyte lineage-specific ablation of *Chd8* affected functional connectivity between several brain regions related to social behavior, and that these changes were correlated with the deficit apparent in the reciprocal social interaction test. Our results thus uncover an apparent relation among brain structural and functional alterations as well as a behavioral change induced by oligodendrocyte dysfunction due to *Chd8* mutation.

## Materials and methods

### Mice

Generation of *Chd8*_*L*_^F/F^ mice was described previously [17]. A pair of loxP sites flanking exons 11 to 13 of *Chd8* in *Chd8*_*L*_^+/F^ mice was deleted by Cre recombinase. *Olig1-Cre* transgenic mice were described previously [31]. *Chd8*_*L*_^+/F^ mice were crossed with *Olig1-Cre* heterozygous mice to produce *Olig1-Cre/Chd8*_*L*_^+/F^ mice. Offspring were backcrossed onto the C57BL/6J line for at least nine generations. All experiments were performed with male mice. Mice were genotyped by polymerase chain reaction-based analysis of genomic DNA with primers for *Chd8* (5′-CCCAAAAGACCAAATCAAACAAAC-3′, 5′-CCATAGGCTGAAGAACCGTAATTG-3′, and 5′-AGGCTTAGAAACCCGTCGAG-3′) and *Cre* (5′-AGGTTCGTTCACTCATGGA-3′ and 5′-TCGACCAGTTTAGTTACCC-3′). All animals were maintained under specific pathogen-free conditions.

### Reciprocal social interaction test

Two mice at 8 to 10 weeks of age and of identical genotypes that had previously been housed in different cages were placed together in a box (40 by 40 by 30 cm) and allowed to explore freely for 10 min (*n* = 12 mice per genotype). Images were captured at a rate of three frames per second. Analysis was performed automatically with the use of ImageSI software, which was developed by Tsuyoshi Miyakawa at Fujita Health University and is based on ImageJ [30, 32].

### rsfMRI analysis in awake mice

rsfMRI was performed on awake mice (*n* = 12 mice per genotype) as previously described [33–36]. Male mice at 6 weeks of age were anesthetized with 2% isoflurane. A custom-made acrylic head bar (3 by 3 by 27 mm) was mounted along the sagittal suture of the exposed skull with the use of dental cement (Super-Bond C&B; Sun Medical, Shiga, Japan). The exposed skull was covered with the dental acrylic, and the animal was returned to its cage and allowed to recover. Mice were acclimated to a mock rsfMRI environment for 2 h per day for at least 10 days before being subjected to rsfMRI in the awake state. Under such conditions, body weight, fecal weight, the electromyogram, and the electrocardiogram of the mice resembled those of unrestrained control mice by the 6th day of habituation [33]. The reciprocal social interaction test was performed before rsfMRI recording with a 7.0-T MRI apparatus equipped with actively shielded gradients at a maximum strength of 700 mT/m (Biospec 70/16; Bruker, Ettlingen, Germany), a cryogenic quadrature radio frequency surface probe (CryoProbe Z120046, Bruker), and ParaVision 6.01 software interface (Bruker). Animal respiration was monitored during the performance of rsfMRI. There was a gap of ~ 1 mm between the top of the head bar holder and the CryoProbe [33]. B0 homogeneity was ensured with the use of automatic iterative FASTMAP methods (ParaVision 6.01). For construction of a reference for brain anatomy, high-resolution T2-weighted images of the whole brain were acquired from mice under anesthesia with 2% isoflurane with the use of a Rapid Acquisition with Relaxation Enhancement (RARE) method and with the following parameters: effective time to echo (TE), 27 ms; time to repetition (TR), 2500 ms; number of averages, 10; RARE factor, 8; matrix size, 182 by 182; field of view (FOV), 12.8 by 12.8; spatial resolution, 0.07 by 0.07 mm; slice thickness, 0.45 mm; number of slices, 30; and slice gap, 0.05 mm. The blood oxygen level-dependent (BOLD) signal was acquired with the use of a gradient echo–echo planar imaging method with the following parameters: TE, 15 ms; TR, 2000 ms; number of averages, 1; segment, 1; band width, 250 kHz; matrix size, 64 by 64; FOV, 12.8 by 12.8; spatial resolution, 0.2 by 0.2 mm; slice thickness, 0.7 mm; number of slices, 20; slice gap, 0.05 mm; flip angle, 70°; and dummy scan, 0. The scan was repeated 300 times in 10 min. Preprocessing and statistical analysis were performed with SPM12 (Welcome Trust Centre for Neuroimaging, London, UK) and in-house software written in MATLAB. Image preprocessing was first performed individually for each animal. The reference images obtained by RARE were segmented with tissue probability maps of gray matter, white matter, and cerebrospinal fluid [37]. Time-series images of BOLD-rsfMRI were realigned to correct for residual head motion and slice timing, and were coregistered to the reference structural images. All images were then spatially normalized and coregistered to the standard structural brain [37]. Finally, the images were resliced to a resolution of 0.2 by 0.2 by 0.2 mm and smoothed with a full width at half maximum (FWHM) Gaussian kernel of 0.6 mm. Functional connectivity was calculated with time-series images of BOLD signals with functional connectivity toolbox (CONN, available at https://www.nitrc.org/projects/conn) and in-house software written in MATLAB. The ART-based scrubbing method in CONN (movement threshold, 2; rotation threshold, 1.15) was used to remove motion artifacts. Signals from white matter and cerebrospinal fluid were also regressed out. Data were detrended and temporally filtered at 0.01 to 0.1 Hz. The global mean signal was regressed out from the preprocessed time-series data in order to reduce global noise and nonneural signal correlations. Analysis of rsfMRI data without global signal regression (GSR) was also performed. Seed-based functional connectivities were computed with the use of regions of interest (ROIs) of 18 bilateral anatomic regions of the cortex divided into left and right hemispheres (MO, SS, AUD, VIS, ACA, PL, ILA, ORB, AI) or 11 bilateral brain loci related to social behavior (PL, ILA, ORB, DG, CA1, SUB, BLA, CP, ACB, VTA, PAG) that were defined by our flexible annotation atlas of the mouse brain based on the Allen Mouse Brain Atlas [38]. Pearson’s correlation coefficients for the time courses of two ROIs were calculated after denoising and despiking and were transformed into Fisher’s z-score. Regions of white matter and cerebrospinal fluid were excluded. An 18 by 18 or 11 by 11 connectivity matrix was then generated for the two groups.

To provide some indication of the overall signal level in our imaging data, we calculated the temporal signal-to-noise ratio (tSNR) of BOLD signal fluctuations as the mean voxel value across 194 time points divided by the temporal standard deviation of the voxel signal [39]. The averaged tSNR value was 70.8 ± 6.6 (*n* = 24), which is similar to that for a previous study [33], suggesting that the loss of tSNR is unlikely in our study.

### DTI acquisition and analysis

The method for DTI acquisition was performed as previously described but with some modifications for the in vivo DTI scans [40, 41]. A DTI-spin echo protocol was used for the acquisition of DTI images of the whole brain of mice (*n* = 12 mice per genotype) under anesthesia with 2% isoflurane with the following parameters: TR, 2800 ms; TE, 17.5 ms; number averaged, 1; segment, 8; band width, 250 kHz; matrix size, 75 by 75; FOV, 13.5 by 13.5 mm; spatial resolution, 0.18 by 0.18 mm; slice thickness, 0.65 mm; number of slices, 21; slice gap, 0.05 mm; b-value, 1000 s/mm^2^; image number with b = 0 s/mm^2^, 6; number of encoding directions of the motion probing gradient, 30; diffusion time δ, 3 ms; Δ, 9 ms; acquisition time, 806 s. The method for DTI analysis was as described previously [40, 41]. The analysis was performed with DSI studio (http://dsi-studio.labsolver.org). A diffusion tensor was modeled at each voxel of the DTI image. Scalar anisotropy and diffusivity maps were obtained from the resulting diffusion tensor eigenvalues (λ1, λ2, λ3), which captured the size of the longest, middle, and shortest axes of the ellipsoid. Fractional anisotropy (FA), radial diffusivity (RD), mean diffusivity (MD), and axial diffusivity (AD) were calculated from standard formulae [42, 43]. An ROI was set with the use of MarsBar (MRC Cognition and Brain Sciences Unit, Cambridge, UK) for significantly altered clusters (voxel-level *P* < 0.01, with a cluster-level false discovery rate [FDR] correction of *P* < 0.05) including MO, SS, acc and pcc, ac, aCP and pCP, CP, ACA, CLA, fx, PALd, fi, and MRN. The same ROI was used to measure FA, RD, MD, and AD values with MarsBar.

### General protocol for behavioral tests and MRI analysis

*Chd8* mutant (*Olig1-Cre/Chd8*_*L*_^+/F^) or control (*Olig1-Cre/Chd8*^+/+^) mice were subjected to the reciprocal social interaction test and MRI analysis on the same day. The procedures were performed in the following sequence: social interaction test, awake rsfMRI analysis, DTI acquisition, and T2-weighted imaging.

### Statistical analysis

All bar graphs show the mean + SEM. Statistical analysis was performed with MATLAB (MathWorks), JMP (SAS Institute, Cary, NC, USA), and Excel (Microsoft, Redmond, WA, USA) software. The Benjamini–Hochberg FDR correction was applied to correct for multiple comparisons within the results of each analysis. Correlations among brain microstructure, functional connectivity, and behavior were evaluated with Pearson’s correlation coefficient and with correction for multiple comparisons. Uncorrected *P* values are shown together with asterisks (*P < 0.05, **P < 0.01, ***P < 0.001) to indicate the significance level after correction by the FDR.

## Results

### Oligodendrocyte lineage-specific *Chd8* heterozygous mutant mice manifest microstructural alterations in the brain

**Fig. 1:**
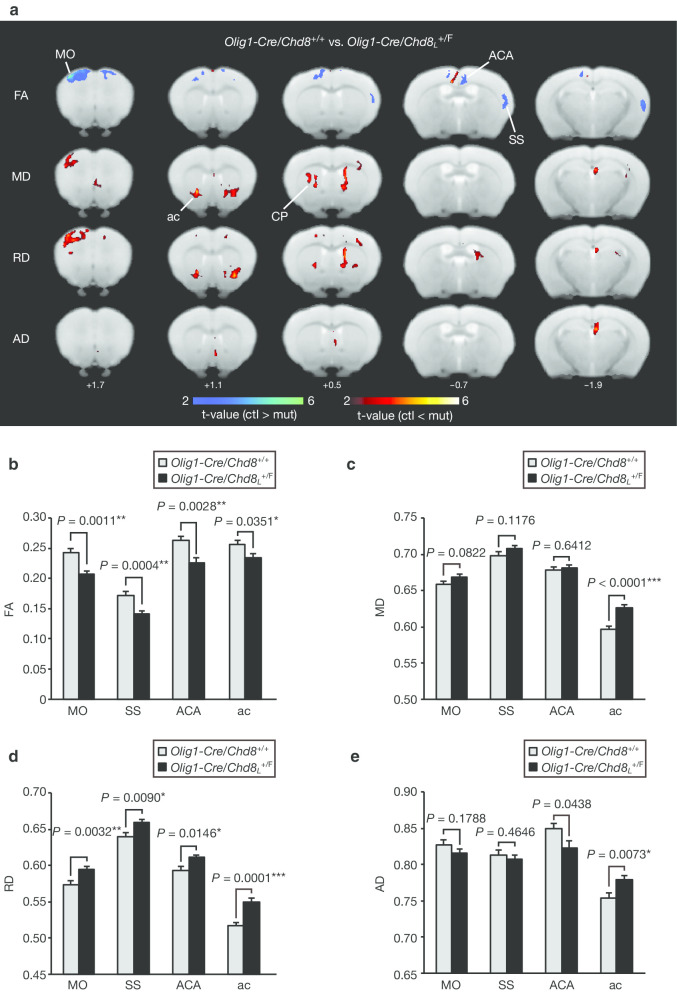
Oligodendrocyte lineage-specific *Chd8* heterozygous mutant mice manifest microstructural changes in the brain. **a** Voxel-based analysis of fractional anisotropy (FA), mean diffusivity (MD), radial diffusivity (RD), and axial diffusivity (AD) in *Olig1-Cre/Chd8*_*L*_^+/F^ mice (mut) compared with *Olig1-Cre/Chd8*^+/+^ mice (ctl) (*n* = 12 mice per genotype). Five rostral-to-caudal axial T2-weighted structural images are overlaid with voxels that showed a significant difference (voxel-level *P* < 0.01, with a cluster-level FDR correction of *P* < 0.05). *MO* motor cortex, *SS* somatosensory cortex, *ACA* anterior cingulate cortex, *CP* caudoputamen, *ac* anterior commissure. **b**–**e** FA (**b**), MD (**c**), RD (**d**), and AD (**e**) values in the indicated regions of interest (ROIs) of *Olig1-Cre/Chd8*_*L*_^+/F^ mice compared with *Olig1-Cre/Chd8*^+/+^ mice (*n* = 12 mice per genotype). Significantly altered clusters of each brain region in (**a**) were defined as ROIs. The ROI “ACA” also included retrosplenial cortex in addition to ACA. The ROI “SS” also included temporal association cortex in addition to SS. The ROI “ac” also included CP and nucleus accumbens (ACB) in addition to ac. Data are means + SEM. Uncorrected *P* values (Student’s *t* test) are shown together with asterisks (*P < 0.05, **P < 0.01, ***P < 0.001) indicating significance level after correction by the FDR. More detailed information for each cluster is provided in Additional file 1: Table S1

We performed DTI analysis to examine microstructural changes in axons and the myelin sheath throughout the brain in oligodendrocyte lineage–specific *Chd8* heterozygous mutant (*Olig1-Cre/Chd8*_*L*_^+/F^) and control (*Olig1-Cre/Chd8*^+/+^) mice. The significantly altered regions of FA, MD, RD, and AD in *Olig1-Cre/Chd8*_*L*_^+/F^ mice relative to control mice were represented in coronal maps (voxel-level *P* < 0.01, with a cluster-level FDR correction of *P* < 0.05) (Fig. 1a and Additional file 1: Table S1). Compared with control mice, *Olig1-Cre/Chd8*_*L*_^+/F^ mice showed a decrease in FA and an increase in MD and RD in the motor cortex (MO), somatosensory cortex (SS), anterior cingulate cortex (ACA), and anterior commissure (ac). The FA values in MO, SS, ACA, and ac of *Olig1-Cre/Chd8*_*L*_^+/F^ mice were significantly lower than those of control mice (Fig. 1b). The MD value in ac, but not those in MO, SS, and ACA, was significantly higher in *Olig1-Cre/Chd8*_*L*_^+/F^ mice than in control animals (Fig. 1c). The RD values in MO, SS, ACA, and ac were significantly higher in *Olig1-Cre/Chd8*_*L*_^+/F^ mice than in control mice (Fig. 1d). Furthermore, the AD value in ac was significantly higher in the mutant mice compared with control mice, whereas those in MO, SS, and ACA did not differ significantly between the two genotypes (Fig. 1e). These results suggested that the microstructure of some regions of the cortex and ac were altered in *Olig1-Cre/Chd8*_*L*_^+/F^ mice.

**Fig. 2:**
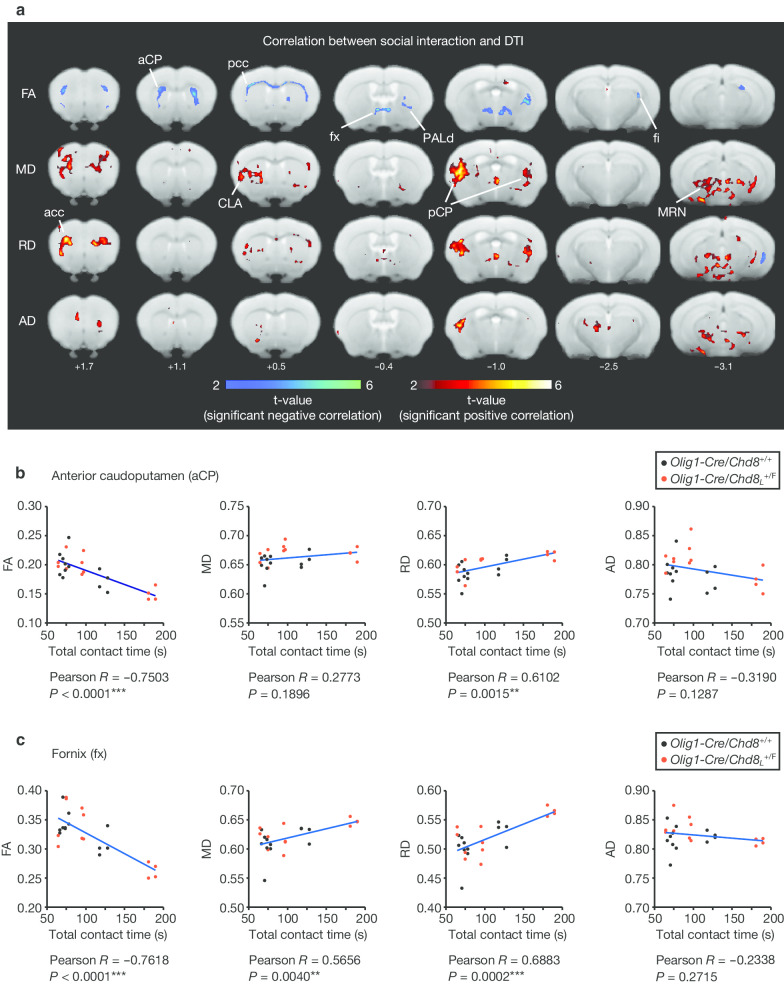
Correlation between microstructural changes in the brain and social behavior in *Olig1-Cre/Chd8*_*L*_^+/F^ mice. **a** Voxel-based comparison between total contact time in the reciprocal social interaction test and FA, MD, RD, or AD values in *Olig1-Cre/Chd8*_*L*_^+/F^ and *Olig1-Cre/Chd8*^+/+^ mice (*n* = 12 mice per genotype). Seven rostral-to-caudal axial T2-weighted structural images are overlaid with voxels that showed a significant correlation (voxel-level *P* < 0.01, with a cluster-level FDR correction of *P* < 0.05). Significant positive and negative correlations between DTI indices and behavior are represented by hot and cold colors, respectively. Two mice of the same genotype that had previously been housed in different cages were placed together in a box and allowed to explore freely for 10 min, after which the mice were subjected to an MRI scan sequence. acc and pcc, anterior and posterior corpus callosum; aCP and pCP, anterior and posterior caudoputamen; fx, fornix; PALd, dorsal pallidum; fi, fimbria; MRN, midbrain reticular nucleus; CLA, claustrum. **b**, **c** Two specific brain regions, aCP (**b**) and fx (**c**), that were associated with total contact time during the reciprocal social interaction test in **a** are highlighted in scatter plots. Significantly altered clusters of each brain region in **a** were defined as ROIs. The ROI “acc” also included motor cortex (MO) in addition to acc. The ROI “fx” also included the anterior commissure (ac) in addition to fx. The ROI “pCP” also included auditory and visceral cortex in addition to pCP. The ROI “PALd” also included CP in addition to PALd. The ROI “CLA” also included somatosensory cortex (SS) and CP in addition to CLA. The ROI “MRN” also included auditory cortex, ectorhinal cortex, and cornu ammonis 1 (CA1) in addition to MRN. Uncorrected *P* values are provided together with asterisks (*P < 0.05, **P < 0.01, ***P < 0.001) indicating significance level after correction by the FDR. Correlation coefficients and statistical values are also provided in Table 1. More detailed information for each cluster and statistical analysis is provided in Additional file 1: Table S1 and Additional file 2: Table S2, respectively

We also evaluated social behavior in *Olig1-Cre/Chd8*_*L*_^+/F^ and control mice before MRI analysis (Additional file 3: Fig. S1), thereby allowing comparison of performance in the reciprocal social interaction test with MRI measurements in the same animals. We previously found that *Olig1-Cre/Chd8*_*L*_^+/F^ mice manifest altered social interaction including an increased contact time during the reciprocal social interaction test [30]. The brain regions showing a significant correlation between total contact time in the behavioral test and DTI indices (FA, MD, RD, and AD) were represented in coronal maps (voxel-level *P* < 0.01, with a cluster-level FDR correction of *P* < 0.05) (Fig. 2a and Additional file 1: Table S1). The FA values in the anterior and posterior corpus callosum (acc and pcc), anterior and posterior caudoputamen (aCP and pCP), fornix (fx), dorsal pallidum (PALd), fimbria (fi), and midbrain reticular nucleus (MRN) were negatively correlated with social interaction (Fig. 2b, c, and Table 1). The RD values in acc, pcc, aCP, pCP, fx, claustrum (CLA), PALd, fi, and MRN were positively correlated with social interaction (Fig. 2b, c, and Table 1). These findings suggested that the structural changes in white matter and striatal pathways were correlated with behavioral change in *Olig1-Cre/Chd8*_*L*_^+/F^ mice.

**Table 1:** Correlation between social interaction and DTI

Structure	Correlation with behavior (*R*)	*P* value
Anterior corpus callosum (acc)		
FA	− 0.6272	0.0001***
MD	0.7353	< 0.0001***
RD	0.8332	< 0.0001***
AD	0.3890	0.0603
Posterior corpus callosum (pcc)		
FA	− 0.7872	< 0.0001***
MD	0.3487	0.0949
RD	0.6438	0.0007**
AD	− 0.4413	0.0309*
Posterior caudoputamen (pCP)		
FA	− 0.6015	0.0019**
MD	0.7701	< 0.0001***
RD	0.7886	< 0.0001***
AD	0.7184	< 0.0001***
Claustrum (CLA)		
FA	− 0.3385	0.1057
MD	0.7457	< 0.0001***
RD	0.7152	< 0.0001***
AD	0.6384	0.0008**
Dorsal pallidum (PALd)		
FA	− 0.7872	< 0.0001***
MD	0.3487	0.0949
RD	0.6438	0.0007**
AD	− 0.4412	0.0309*
Fimbria (fi)		
FA	− 0.7413	< 0.0001***
MD	0.1210	0.5733
RD	0.4421	0.0305*
AD	− 0.3079	0.1432
Midbrain reticular nucleus (MRN)		
FA	− 0.4576	0.0246*
MD	0.7908	< 0.0001***
RD	0.7821	< 0.0001***
AD	0.7851	< 0.0001***

Pearson’s correlation coefficient (*R*) and *P* value for total contact time in the reciprocal social interaction test and FA, MD, RD, or AD values in the indicated brain regions are shown. Uncorrected *P* values are provided together with asterisks (*P < 0.05, **P < 0.01, ***P < 0.001) indicating significance level after correction by the FDR. Correlation coefficients and statistical values are also provided in Additional file 2: Table S2

### Oligodendrocyte lineage-specific *Chd8* heterozygous mutant mice manifest altered functional brain connectivity

**Fig. 3:**
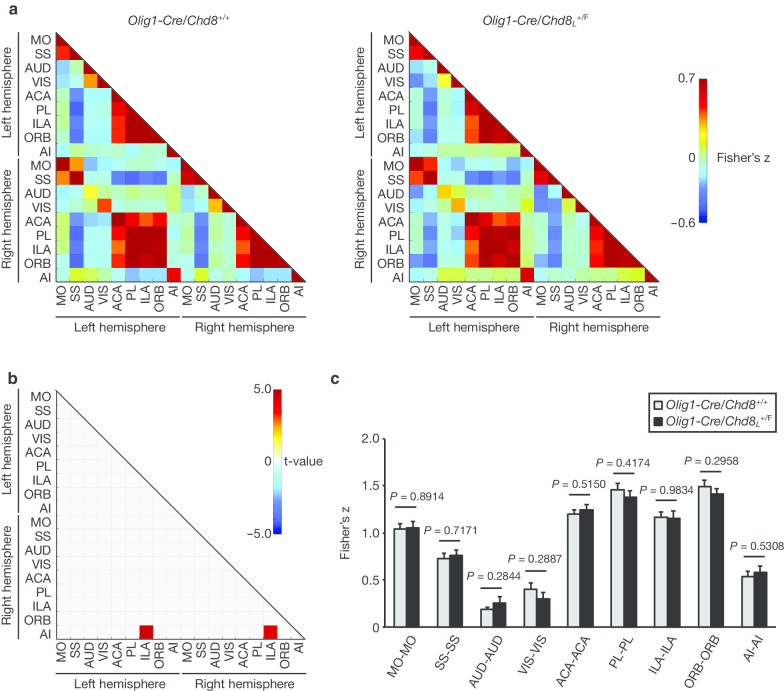
Contralateral connectivity in *Olig1-Cre/Chd8*_*L*_^+/F^ and control mice. **a** Correlation matrices derived from rsfMRI BOLD signal analysis for connections between left and right hemispheres of the cerebral cortex (*n* = 12 mice per genotype). *MO* motor cortex, *SS* somatosensory cortex, *AUD* auditory cortex, *VIS* visual cortex, *ACA* anterior cingulate cortex, *PL* prelimbic cortex, *ILA* infralimbic cortex, *ORB* orbitofrontal cortex, *AI* agranular insular cortex. **b** The t-value for significantly altered contralateral regional connectivity of *Olig1-Cre/Chd8*_*L*_^+/F^ mice compared with *Olig1-Cre/Chd8*^+/+^ mice (FDR-adjusted *P* value of < 0.05). **c** Strength of the correlation (Fisher’s z-score) between the indicated brain regions of *Olig1-Cre/Chd8*_*L*_^+/F^ and *Olig1-Cre/Chd8*^+/+^ mice (*n* = 12 mice per genotype). Data are means + SEM. *P* values were determined with Student’s *t* test. The results of rsfMRI processing without GSR are provided in Additional file 3: Fig. S2

Given that altered inter- and intrahemispheric functional connectivity has been detected in individuals with ASD [44], we next assessed long-range functional brain connectivity by measurement of the correlation of BOLD signals across brain regions in awake *Olig1-Cre/Chd8*_*L*_^+/F^ and control mice with rsfMRI. Functional connectivity between pairs of cortical ROIs was compared between *Olig1-Cre/Chd8*_*L*_^+/F^ and control mice. ROIs of 18 bilateral anatomic regions in the left and right hemispheres of the cortex were defined from our flexible annotation atlas based on the Allen Brain Atlas [38], and we constructed correlation matrices of functional connectivity between these ROIs in *Olig1-Cre/Chd8*_*L*_^+/F^ and control mice (Fig. 3a). Whereas the extent of functional connectivity between right agranular insular cortex (AI) and left or right infralimbic cortex (ILA) was significantly increased in *Olig1-Cre/Chd8*_*L*_^+/F^ mice compared with control mice (Fig. 3b), the extent of connectivity of corresponding ROIs between the right and left hemispheres did not differ significantly between the two genotypes (Fig. 3c). To examine the possible effect of GSR on these results, we also analyzed rsfMRI data without such regression. Without GSR, we did not detect any significant differences between the two genotypes with regard to the extent of functional connectivity between the right and left hemispheres (Additional file 3: Fig. S2).

**Fig. 4:**
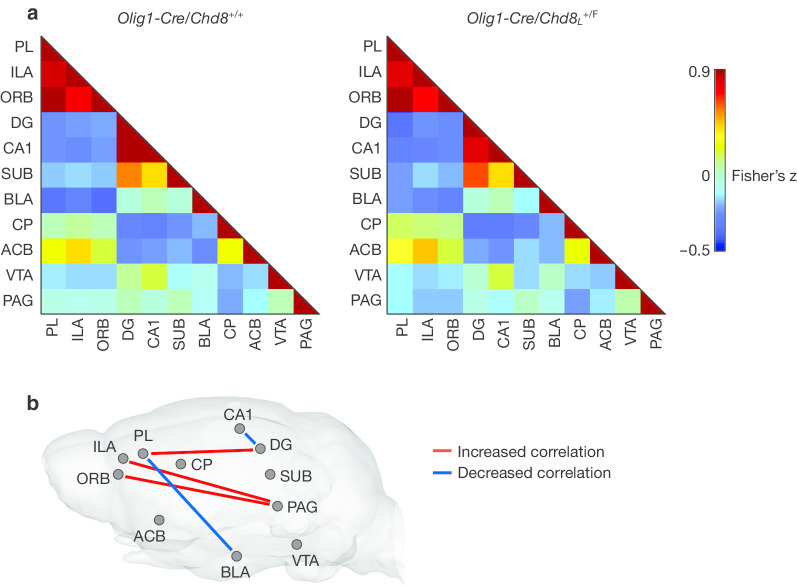
Oligodendrocyte lineage–specific *Chd8* heterozygous mutant mice manifest altered functional brain connectivity. **a** Correlation (Fisher’s z-score) matrices derived from rsfMRI BOLD signal analysis for connections between brain regions related to ASD in *Olig1-Cre/Chd8*_*L*_^+/F^ and *Olig1-Cre/Chd8*^+/+^ mice (*n* = 12 mice per genotype). *PL* prelimbic cortex, *ILA* infralimbic cortex, *ORB* orbitofrontal cortex, *DG* dentate gyrus, *CA1* cornu ammonis 1, *SUB* subiculum, *BLA* basolateral amygdala, *CP* caudoputamen, *ACB* nucleus accumbens, *VTA* ventral tegmental area, *PAG* periaqueductal gray. **b**, Significant changes in the strength (absolute value of the correlation coefficient) of functional connectivity between the indicated brain regions of *Olig1-Cre/Chd8*_*L*_^+/F^ versus *Olig1-Cre/Chd8*^+/+^ mice (*n* = 12 mice per genotype). The significantly altered connections (Student’s *t* test) in the mutant mice are represented by red (increased absolute correlation) or blue (decreased absolute correlation) lines in a brain depiction, although these differences did not survive multiple comparison correction. The results of rsfMRI processing without GSR are provided in Additional file 3: Fig. S3

To investigate further the effects of oligodendrocyte lineage-specific *Chd8* heterozygous mutation on functional connectivity, we evaluated the connectivity between prelimbic cortex (PL), ILA, orbitofrontal cortex (ORB), dentate gyrus (DG), cornu ammonis 1 (CA1), subiculum (SUB), basolateral amygdala (BLA), CP, nucleus accumbens (ACB), ventral tegmental area (VTA), and periaqueductal gray (PAG), all of which are related to social behavior [45, 46]. We constructed correlation matrices of functional connectivity between these ROIs in *Olig1-Cre/Chd8*_*L*_^+/F^ and control mice (Fig. 4a). The correlation between PL and DG, ILA and PAG, or ORB and PAG was increased in *Olig1-Cre/Chd8*_*L*_^+/F^ mice, whereas the negative correlation between PL and BLA was diminished in *Olig1-Cre/Chd8*_*L*_^+/F^ mice compared with control mice (Fig. 4b). We also found that the extent of functional connectivity between DG and CA1 was reduced in *Olig1-Cre/Chd8*_*L*_^+/F^ mice compared with control mice (Fig. 4b). Although these differences did not survive multiple corrections, uncorrected statistics supported the alterations of functional connectivity in *Olig1-Cre/Chd8*_*L*_^+/F^ mice. Similar results were obtained without GSR for the correlation between ORB and PAG and between PL and BLA (Additional file 3: Fig. S3). To evaluate the possible effect of head motion on functional connectivity, we calculated the framewise displacement from six parameters of head motion, which were estimated during the realignment process [47], and then examined the relation between framewise displacement and functional connectivity. Functional connectivity between PL and BLA did not show a significant correlation with head motion (Additional file 3: Fig. S4), suggesting that head motion during the acquisition of rsfMRI data did not affect the functional connectivity values. These results thus suggested that functional brain connectivity related to social behavior was altered in *Olig1-Cre/Chd8*_*L*_^+/F^ mice.

### Altered functional brain connectivity is correlated with structural and behavioral changes

**Fig. 5:**
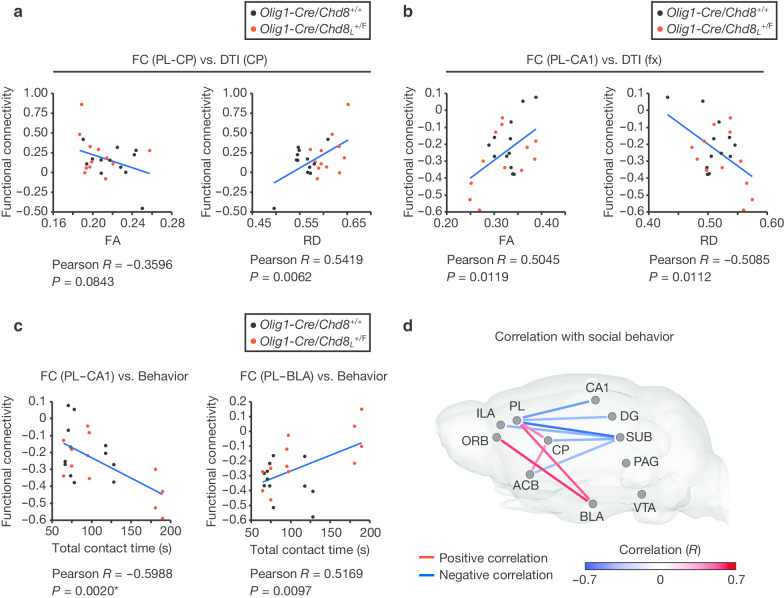
Correlation of altered functional brain connectivity with brain structural and behavioral changes. **a**, **b** Correlation between the strength of functional connectivity (FC) for PL-CP (**a**) or PL-CA1 (**b**) and FA or RD values in CP (**a**) or fx (**b**) of *Olig1-Cre/Chd8*_*L*_^+/F^ and *Olig1-Cre/Chd8*^+/+^ mice (*n* = 12 mice per genotype). **c** Correlation between total contact time in the reciprocal social interaction test and the strength of functional connectivity for PL-CA1 or PL-BLA. Pearson’s correlation coefficient (*R*) and uncorrected *P* values are shown, with * indicating *P* < 0.05 after correction by FDR. **d** Positive and negative correlations between the duration of social interaction and the strength of functional connectivity between the indicated brain regions are represented by red and blue lines, respectively (color shade reflects the correlation coefficient *R*). *PL* prelimbic cortex, *ILA* infralimbic cortex, *ORB* orbitofrontal cortex, *DG* dentate gyrus, *CA1* cornu ammonis 1, *SUB* subiculum, *BLA* basolateral amygdala, *CP* caudoputamen, *ACB* nucleus accumbens, *VTA* ventral tegmental area, *PAG* periaqueductal gray. The correlation between the extent of functional connectivity in PL-CA1, PL-SUB, and ORB-BLA and social interaction survived FDR corrections. Correlation coefficients and statistical values are also provided in Table 2 and Additional file 2: Table S2. The results of rsfMRI processing without GSR are provided in Additional file 3: Fig. S5

We examined whether the structural changes detected in the brain of *Olig1-Cre/Chd8*_*L*_^+/F^ mice were associated with the extent of functional connectivity. The medial prefrontal cortex (mPFC) including PL and ILA projects to the dorsomedial portions of CP in rodents [48], and structural changes in CP were apparent in *Olig1-Cre/Chd8*_*L*_^+/F^ mice (Fig. 1). The RD value in CP was positively correlated with the extent of functional connectivity between PL and CP in *Olig1-Cre/Chd8*_*L*_^+/F^ and control mice, although these differences did not survive multiple corrections (Fig. 5a). We also detected a correlation of functional connectivity between several other regions with structural changes in CP (Table 2). The fx is the major association pathway connecting the hippocampus with other regions including mPFC [49], and we detected a correlation between structural changes of fx and social interaction (Fig. 2c). We also found that FA and RD values in fx were correlated with the extent of functional connectivity between PL and CA1 among several other brain regions (Fig. 5b and Table 2).

**Table 2:** Correlation between functional connectivity and DTI or social interaction

DTI or behavior	Functional connectivity	Correlation (*R*)	*P* value
FA (CP)	ILA–ACB	− 0.4186	0.0417
RD (CP)	PL–DG	− 0.4104	0.0464
	PL–ACB	0.5516	0.0052
	PL–CP	0.5419	0.0062
	ILA–CA3	− 0.4141	0.0442
	ILA–CP	0.4489	0.0278
	ILA–ACB	0.5682	0.0038
	ORB–CA3	− 0.4169	0.0427
	ORB–CP	0.5126	0.0104
	ORB–ACB	0.4751	0.0190
FA (fx)	PL–DG	0.4286	0.0367
	PL–CA1	0.5045	0.0119
	PL–SUB	0.5281	0.0080
	PL–BLA	− 0.4092	0.0471
	PL–CP	− 0.4796	0.0177
	ILA–SUB	0.4670	0.0214
	ACB–CP	− 0.4226	0.0397
RD (fx)	PL–CA1	− 0.5085	0.0112
	PL–SUB	− 0.4442	0.0297
	PL–CP	0.5907	0.0024
	PL–ACB	0.5339	0.0072
	ILA–SUB	− 0.4218	0.0400
	ACB–CP	0.4477	0.0282
Behavior (total contact time)	PL–DG	− 0.5065	0.0116
	PL–CA1	− 0.5988	0.0020*
	PL–SUB	− 0.6877	0.0002**
	PL–BLA	0.5169	0.0097
	PL–CP	0.4333	0.0344
	ILA–SUB	− 0.5230	0.0077
	ORB–BLA	0.5742	0.0033*
	CP–SUB	− 0.5240	0.0086
	CP–ACB	0.4091	0.0472
	ACB–SUB	− 0.4798	0.0177

Pearson’s correlation coefficient (*R*) and *P* value for functional connectivity between the indicated brain regions and either FA or RD in the indicated brain regions or total contact time in the reciprocal social interaction test are shown. *PL* prelimbic cortex, *ILA* infralimbic cortex, *ORB* orbitofrontal cortex, *DG* dentate gyrus, *CA1* cornu ammonis 1, *SUB* subiculum, *BLA* basolateral amygdala, *CP* caudoputamen, *ACB* nucleus accumbens. Uncorrected *P* values are provided together with asterisks (*P < 0.05, **P < 0.01) indicating significance level after correction by the FDR. Correlation coefficients and statistical values are also provided in Additional file 2: Table S2

We finally investigated whether altered functional connectivity in *Olig1-Cre/Chd8*_*L*_^+/F^ mice was correlated with altered social interaction. The extent of functional connectivity between PL and the hippocampus including CA1, DG, and SUB was negatively correlated with social interaction, whereas the extent of functional connectivity between BLA and either PL or ORB was positively correlated with social interaction (Fig. 5c, d). We also detected a significant correlation between the extent of functional connectivity between several other regions (with or without GSR) and total interaction time during the reciprocal social interaction test (Fig. 5d, Table 2, and Additional file 3: Fig. S5). Among these results, the correlation between the extent of functional connectivity in PL-CA1, PL-SUB, and ORB-BLA and social interaction survived FDR corrections. Examination of the relation between functional connectivity and social interaction within the same genotype revealed that almost all significant correlations observed in the analysis of both genotypes were also apparent in *Olig1-Cre*/*Chd8*_*L*_^+/F^ mice (Additional file 2: Table S2). These results suggested that altered functional connectivity in *Olig1-Cre/Chd8*_*L*_^+/F^ mice is associated with both brain structural and behavioral changes.

## Discussion

Alterations in brain microstructure and functional connectivity are common characteristics of individuals with ASD as well as of mouse models of this disorder. To investigate which brain regions are altered in association with specific genetic mutations and whether these alterations contribute to autism-related behavior is key to understanding the etiology of ASD. We have now shown that oligodendrocyte-specific ablation of *Chd8* results in changes to morphology and functional connectivity across the brain. We also found that altered functional connectivity between several brain regions was correlated with structural and behavioral changes.

Altered social interaction is a prominent characteristic of ASD. Three independent groups have reproducibly found that *Chd8* heterozygous knockout mice manifest an increased contact time during the reciprocal social interaction test as well as altered social novelty preference during the three-chamber test [17, 18, 20]. Although increased contact time is not a typical behavioral characteristic of other ASD model mice, it may be a characteristic of social behavior in *Chd8* mutant mice. Given that *Chd8* mutation restricted to cells of the oligodendrocyte lineage recapitulated this behavioral phenotype [30], this mouse model appears to be well suited to analysis of functional brain connectivity underlying social behavior. *Olig1-Cre*/*Chd8*_*L*_^+/F^ mice in the present study did not show an increased contact time in the reciprocal social interaction test (Additional file 3: Fig. S1), probably because of differences related to sample size, head bar attachment, surgery, habituation procedure, and environmental factors. However, our results have revealed that changes in brain structure or functional connectivity are correlated with social interaction in *Olig1-Cre/Chd8*_*L*_^+/F^ mice.

Our DTI data revealed an increase in RD values and a reduction in FA values in MO, SS, ACA, and ac in *Olig1-Cre/Chd8*_*L*_^+/F^ mice compared with control mice. Whereas FA is a summary measure of microstructural changes, RD reflects the structural status of myelin as well as axons [41]. Given that both *Chd8* heterozygous mutant mice and *Olig1-Cre/Chd8*_*L*_^+/F^ mice manifest a reduced thickness of the myelin sheath [30], the altered FA and RD values of *Olig1-Cre/Chd8*_*L*_^+/F^ mice might be attributable to myelination defects in these animals. It is of note that regions showing microstructural changes in *Olig1-Cre/Chd8*_*L*_^+/F^ mice include the corticostriatal pathway, which plays a role in cognition, motivation, and reward processing [50] and is implicated in ASD pathogenesis [51]. Striatal dysfunction has thus previously been demonstrated in *Chd8* heterozygous mutant mice [18]. Defects in synaptic transmission by medium spiny neurons have also been detected in *Chd8* heterozygous mutant mice, and *Chd8* ablation in ACB, but not in the dorsal striatum, was found to facilitate acquired motor learning [18]. Microstructural changes due to oligodendrocyte defects might thus contribute to changes in neural activity and behavior in these mice. Although we have previously shown with the use of electron microscopy that the myelin sheath is thinner in cc of *Olig1-Cre/Chd8*_*L*_^+/F^ mice [30], we did not detect any significant differences in FA or RD values in cc, likely as a result of the relatively small effect of *Chd8* mutation on myelination or of technical limitations such as those related to the resolution or signal-to-noise ratio of DTI. We previously showed that myelination defects are present throughout the brain of *Chd8* mutant mice including cortical regions [30], suggesting that myelination is also impaired in the regions that show alterations by DTI in the present study. Structural alterations in cc that are correlated with behavioral changes may be indicative of mild axonal or myelination defects.

The performance of behavioral analysis before MRI allowed us to assess the correlation between behavioral phenotype and structural or functional changes in the brain. Abnormal social behavior is the most prominent phenotype of individuals with ASD and is also observed in both *Chd8* heterozygous mutant mice and *Olig1-Cre/Chd8*_*L*_^+/F^ mice [17, 18, 20, 30]. Consistent with the previous finding that FA in cc and fx was negatively correlated with autism symptom severity as measured by the Autism Diagnostic Interview-Revised (ADI-R) in individuals with ASD [3], we found that FA and RD values in several brain regions including cc and fx were correlated with total contact time in the reciprocal social interaction test in *Olig1-Cre/Chd8*_*L*_^+/F^ and control mice. Whereas microstructural changes in the cortex and striatum were thus detected in *Olig1-Cre/Chd8*_*L*_^+/F^ mice, it remains unclear whether these changes are attributable to alterations in axons and dendrites in addition to myelination defects. Further analysis will be required to identify the mechanisms underlying such structural changes in several brain regions.

Action potential propagation is delayed as a result of myelination defects in *Olig1-Cre/Chd8*_*L*_^+/F^ mice [30]. However, it had remained unclear whether this delay affects neural networks in the brain of these mice. Given that *Chd8* mutation results in a reduced conduction velocity in the cc connecting the left and right hemispheres [30], we investigated interhemispheric functional connectivity in *Olig1-Cre*/*Chd8*_*L*_^+/F^ mice. However, we detected little effect of such *Chd8* mutation on inter- or intrahemispheric functional connectivity, suggesting that the reduction in conduction velocity in the cc of *Olig1-Cre*/*Chd8*_*L*_^+/F^ mice is not sufficient to influence functional connectivity in the examined regions. In contrast, we found that synchronization of rsfMRI signals between several brain regions related to ASD was altered in the mutant mice. Changes in functional connectivity in the mPFC, hippocampus, and amygdala were recently detected in individuals with ASD from four independent cohorts [52], with the amygdala having previously been shown to play a key role in social behavior and emotional information processing [53, 54]. The amygdala has reciprocal connections with multiple brain areas including the prelimbic prefrontal cortex, and our rsfMRI data showed that alterations in BLA connectivity in oligodendrocyte lineage-specific *Chd8* heterozygous mutant mice were significantly correlated with total contact time in the reciprocal social interaction test. We also found that functional connectivity between PL and CA1 was associated with alterations in FA in fx and the behavioral deficit in the mutant mice. Excitatory neurons in the ventral hippocampus project to mPFC through fx, and these connections are related to social behavior [55, 56]. We speculate that delayed action potential propagation due to a myelination defect in fx might affect postsynaptic responses in mPFC and thereby give rise to altered social interaction in *Olig1-Cre/Chd8*_*L*_^+/F^ mice. It is also possible that compensatory changes as a result of oligodendrocyte dysfunction during development might influence behavioral characteristics of *Olig1-Cre*/*Chd8*_*L*_^+/F^ mice. For example, Nogo receptor ligand, a myelin glycoprotein expressed in oligodendrocytes, inhibits neurite outgrowth [57]. It remains to be determined whether behavioral deficits in *Olig1-Cre*/*Chd8*_*L*_^+/F^ mice depend on myelination defects or are secondary effects of oligodendrocyte dysfunction.

We applied GSR to eliminate undesired global noise and bias and to detect negative correlation. We performed awake rsfMRI, and movement-related noise is thought to be eliminated with the use of GSR. We were able to detect many changes in functional connectivity. We believe that the results obtained with GSR are thus more reliable in the present study. However, given the possibility that differences might be diminished with the use of GSR [58], we also provide the results of functional connectivity analysis without GSR (Additional file 3: Figs. S2, S3, and S5).

Whereas *Chd8* heterozygous mutant mice were previously found to manifest increased connectivity in entorhinal, retrosplenial, and auditory cortical as well as posterior hippocampal areas [20], we detected no such alterations in these brain regions of *Olig1-Cre*/*Chd8*_*L*_^+/F^ mice. This difference might be explained by a difference in affected cell types of the mutant mice (*Chd8*^+/−^ versus *Olig1-Cre*/*Chd8*_*L*_^+/F^ mice) or in experimental conditions (anesthetized or awake mice during rsfMRI). Furthermore, recent studies with various mouse models of ASD have detected changes in functional connectivity in many brain regions including PFC, MO, SS, ACA, AI, retrosplenial cortex, ventral hippocampus, CP, amygdala, hindbrain, thalamic nuclei, and medial-dorsal thalamus [24–29]. These ASD model mice as well as our mice overlap to only a small extent in their signatures of affected brain networks. Further studies will be necessary to investigate the causal relation between altered functional connectivity and ASD-like behaviors, as well as to elucidate how each genetic mutation gives rise to such connectivity changes in individuals with ASD or in model animals. Collectively, our results indicate that oligodendrocyte dysfunction induced by *Chd8* mutation influences neuroanatomic and functional connectivity throughout the brain. They may thus be of translational value for further ASD research as well as provide insight into neural mechanisms underlying ASD.

## Supplementary information

**Additional file 1: Table S1.** This file contains detailed information for clusters in DTI analysis.

**Additional file 2: Table S2.** This file contains raw data and detailed information for correlation analysis.

**Additional file 3: Figures S1–S5.** This file contains additional figures.

## Data Availability

The data sets used and/or analyzed during the current study are available from the corresponding author on reasonable request.

## Abbreviations

ac: Anterior commissure
ACA: Anterior cingulate cortex
ACB: Nucleus accumbens
acc: Anterior corpus callosum
aCP: Anterior caudoputamen
AD: Axial diffusivity
AI: Agranular insular cortex
ASD: Autism spectrum disorder
AUD: Auditory cortex
BLA: Basolateral amygdala
BOLD: Blood oxygen level-dependent
CA: Cornu ammonis
CHD8: Chromodomain helicase DNA-binding protein 8
CLA: Claustrum
CP: Caudoputamen
DG: Dentate gyrus
DTI: Diffusion tensor imaging
FA: Fractional anisotropy
FDR: False discovery rate
fi: Fimbria
FOV: Field of view
fx: Fornix
GSR: Global signal regression
ILA: Infralimbic cortex
MD: Mean diffusivity
MO: Motor cortex
mPFC: Medial prefrontal cortex
MRI: Magnetic resonance imaging
MRN: Midbrain reticular nucleus
ORB: Orbitofrontal cortex
PAG: Periaqueductal gray
PALd: Dorsal pallidum
pcc: Posterior corpus callosum
pCP: Posterior caudoputamen
PL: Prelimbic cortex
RARE: Relaxation enhancement
RD: Radial diffusivity
ROI: Region of interest
rsfMRI: Resting-state functional MRI
SS: Somatosensory cortex
SUB: Subiculum
TE: Effective time to echo
TR: Time to repetition
tSNR: Temporal signal-to-noise ratio
VIS: Visual cortex
VTA: Ventral tegmental area
