# Supplementary material for: Chd8 mutation in oligodendrocytes alters microstructure and functional connectivity in the mouse brain
Source: Mol Brain. 2020 Nov 23;13:160. doi: 10.1186/s13041-020-00699-x (PMC7686671; doi:10.1186/s13041-020-00699-x)
Supplement: Supplementary file 3 — Additional file 3: Figures S1–S5. This file contains additional figures. [file 13041_2020_699_MOESM3_ESM.pdf]

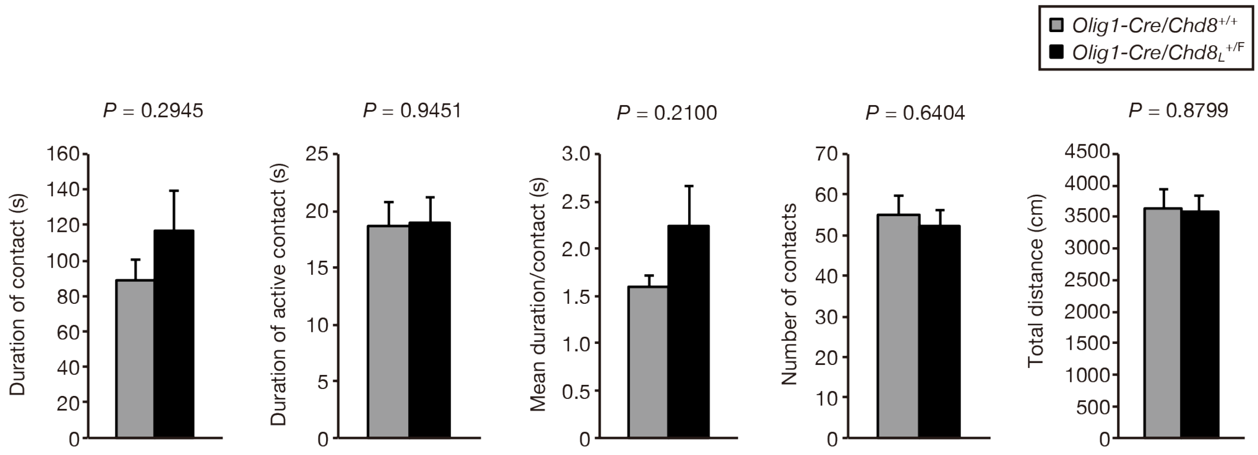

Fig. S1 Reciprocal social interaction test for *Olig1-Cre/*$Chd8_L^{+/F}$ and control mice. Total duration of contacts, total duration of active contacts, mean duration of contacts, number of contacts, and total distance traveled are shown [n = 12 mice (6 pairs) per genotype]. Two male mice of the same genotype at 8 to 10 weeks of age that had previously been housed in different cages were placed together in a box and allowed to explore freely for 10 min. Data are means + SEM. P values were determined by Student's t test.

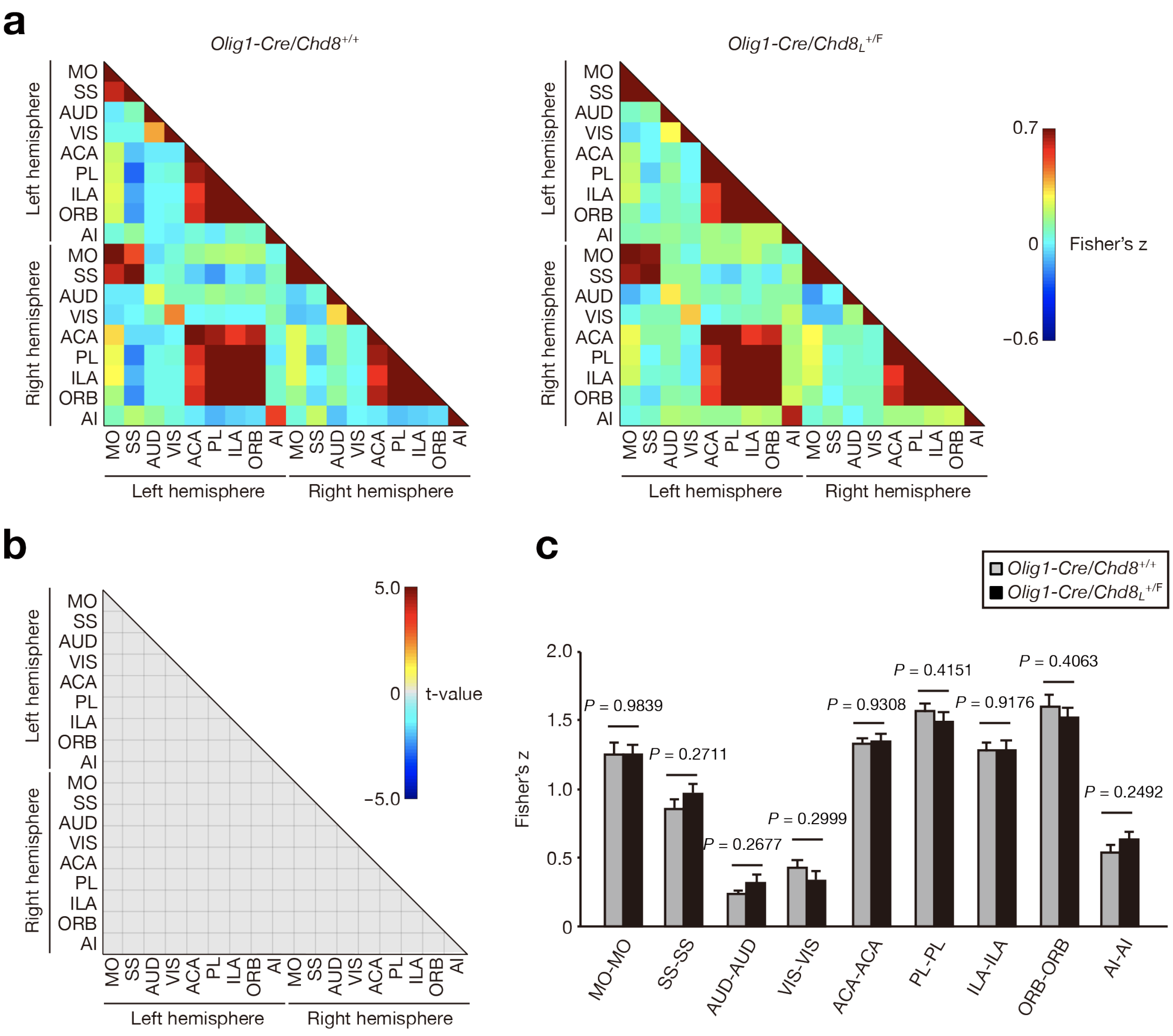

Fig. S2 Contralateral connectivity in *Olig1-Cre/Chd8*$_L$$^{+/F}$ and control mice without GSR. **a** Correlation matrices derived from rsfMRI BOLD signal analysis without GSR for connections between left and right hemispheres of the cerebral cortex (*n* = 12 mice per genotype). MO, motor cortex; SS, somatosensory cortex; AUD, auditory cortex; VIS, visual cortex; ACA, anterior cingulate cortex; PL, prelimbic cortex; ILA, infralimbic cortex; ORB, orbitofrontal cortex; AI, agranular insular cortex. **b** The t-value for significantly altered contralateral regional connectivity of *Olig1-Cre/Chd8*$_L$$^{+/F}$ mice compared with *Olig1-Cre/Chd8*$^{+/+}$ mice (FDR-adjusted *P* value of <0.05). **c**, Strength of the correlation (Fisher's z-score) between the indicated brain regions of

Olig1-Cre/Chd8$_L^{+/F}$ and *Olig1-Cre/Chd8$^{+/+}$* mice (n = 12 mice per genotype). Data are means + SEM. P values were determined with Student's t test.

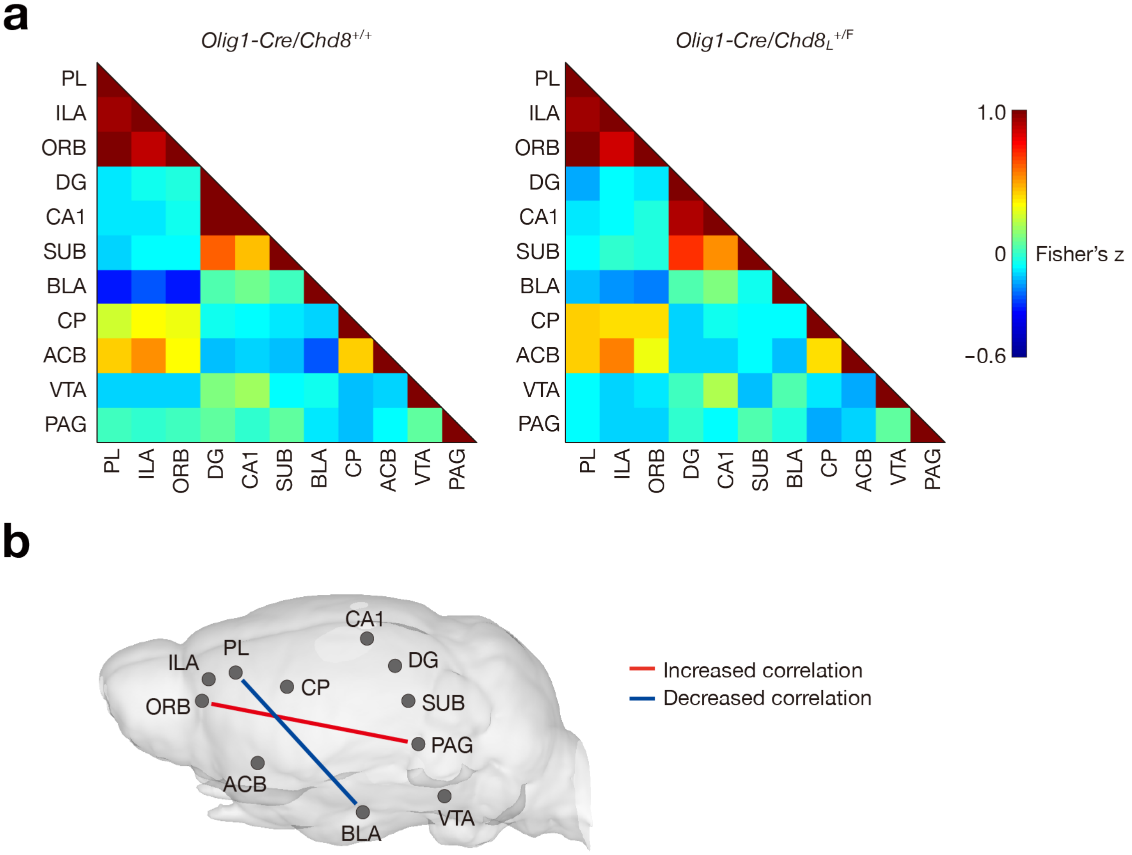

Fig. S3 Oligodendrocyte lineage–specific *Chd8* heterozygous mutant mice manifest altered functional brain connectivity. **a** Correlation (Fisher's z-score) matrices derived from rsfMRI BOLD signal analysis without GSR for connections between brain regions related to ASD in *Olig1-Cre/Chd8*$_L^{+/F}$ and *Olig1-Cre/Chd8*$^{+/+}$ mice (n = 12 mice per genotype). PL, prelimbic cortex; ILA, infralimbic cortex; ORB, orbitofrontal cortex; DG, dentate gyrus; CA1, cornu ammonis 1; SUB, subiculum; BLA, basolateral amygdala; CP, caudoputamen; ACB, nucleus accumbens; VTA, ventral tegmental area; PAG, periaqueductal gray. **b** Significant changes in the strength (absolute value of the correlation coefficient) of functional connectivity between the indicated brain regions of *Olig1-Cre/Chd8*$_L^{+/F}$ versus *Olig1-Cre/Chd8*$^{+/+}$ mice (n = 12 mice per genotype). The significantly altered connections (Student's t test) in the mutant mice are represented by red (increased absolute correlation) or blue (decreased absolute correlation) lines in a brain depiction, although these differences did not survive multiple comparison correction.

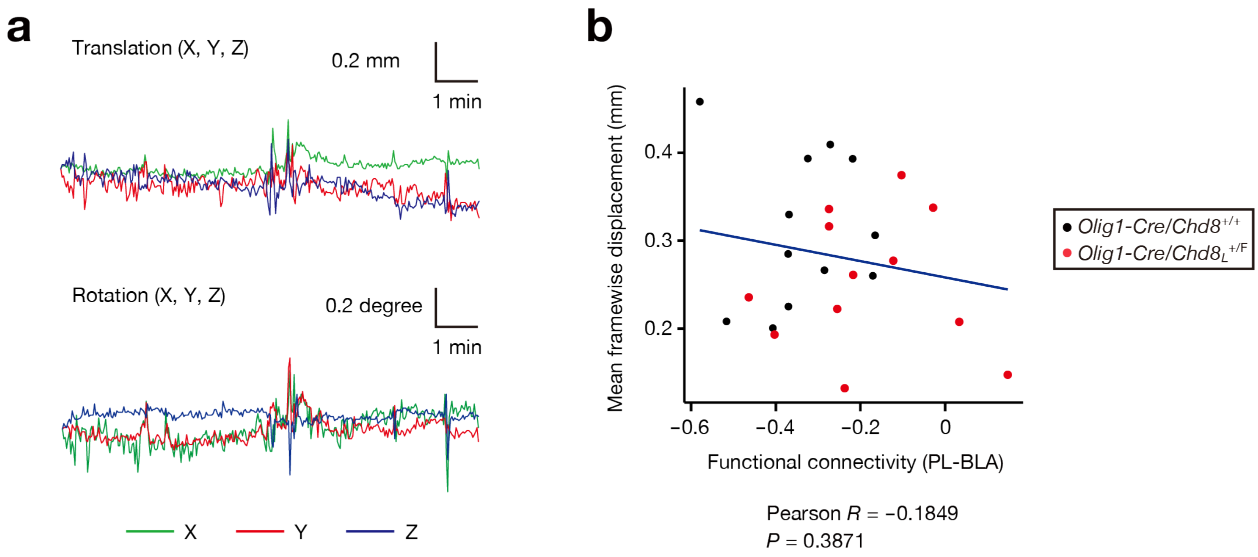

Fig. S4 Effect of head motion on rsfMRI. **a** Representative time course data for six parameters (translational and rotational motion) calculated for frame-by-frame realignment of rsfMRI. **b** Plots of averaged functional connectivity between prelimbic cortex (PL) and basolateral amygdala (BLA) and averaged framewise displacement, an index of head motion in each mouse, indicated that rsfMRI was not dependent on head motion (Pearson's correlation).

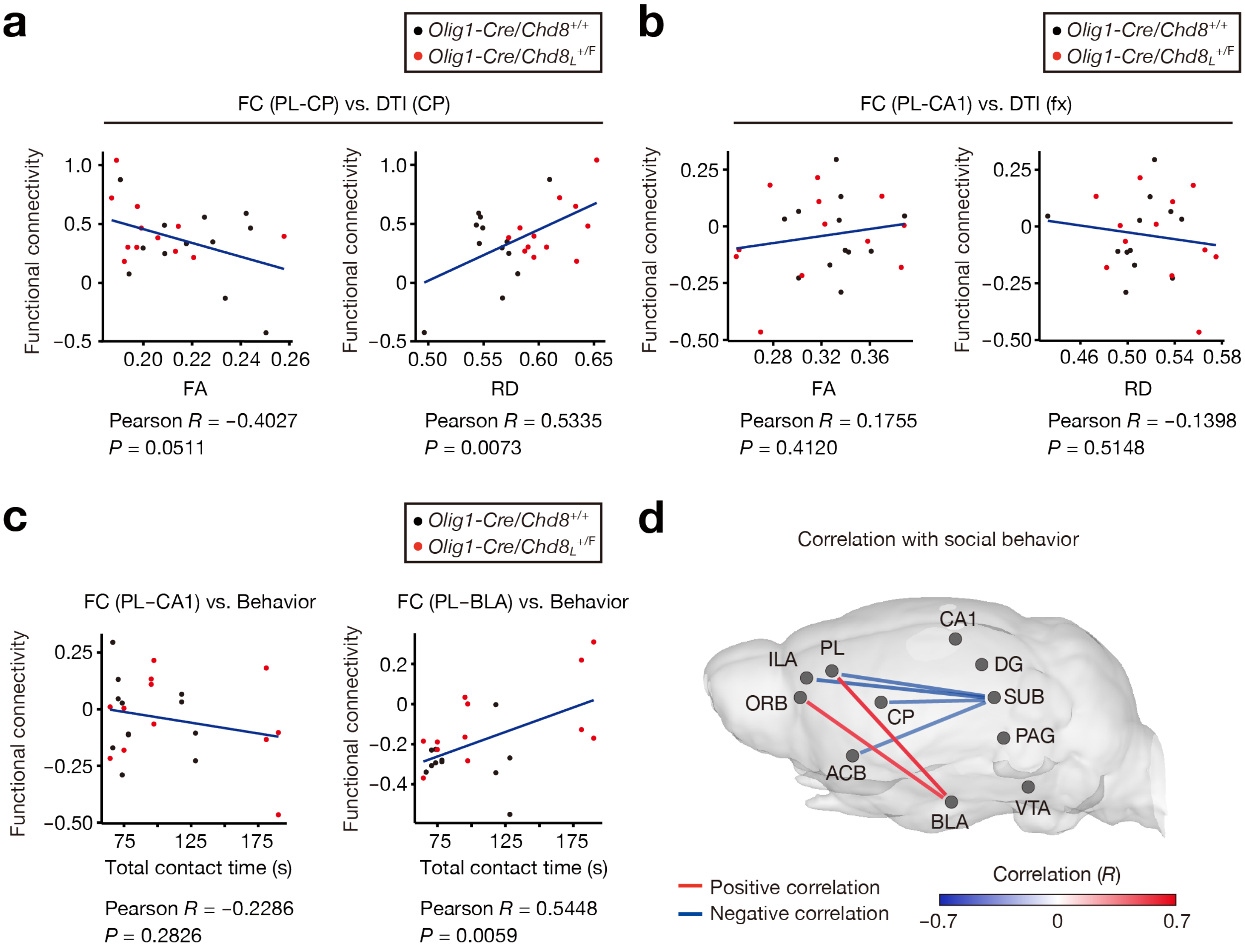

Fig. S5 Correlation of altered functional brain connectivity with brain structural and behavioral changes. **a**, **b** Correlation between the strength of functional connectivity (FC) without GSR for PL-CP (**a**) or PL-CA1 (**b**) and FA or RD values in CP (**a**) or fx (**b**) of *Olig1-Cre/Chd8*$_L^{+/F}$ and *Olig1-Cre/Chd8*$^{+/+}$ mice (n = 12 mice per genotype). **c**, Correlation between total contact time in the reciprocal social interaction test and the strength of functional connectivity for PL-CA1 or PL-BLA. Pearson's correlation coefficient (R) and uncorrected P values are shown. **d** Positive and negative correlations between the duration of social interaction and the strength of functional connectivity without GSR between the indicated brain regions are represented by red and blue lines, respectively (color shade reflects the correlation coefficient R). These differences did not survive multiple comparison correction. PL, prelimbic cortex; ILA, infralimbic cortex; ORB,

orbitofrontal cortex; DG, dentate gyrus; CA1, cornu ammonis 1; SUB, subiculum; BLA, basolateral amygdala; CP, caudoputamen; ACB, nucleus accumbens; VTA, ventral tegmental area; PAG, periaqueductal gray.
